# Genome analysis provides insights into microaerobic toluene-degradation pathway of *Zoogloea oleivorans* Buc^T^

**DOI:** 10.1007/s00203-019-01743-8

**Published:** 2019-10-28

**Authors:** András Táncsics, Milán Farkas, Balázs Horváth, Gergely Maróti, Lauren M. Bradford, Tillmann Lueders, Balázs Kriszt

**Affiliations:** 1 Regional University Center of Excellence in Environmental Industry, Szent István University, Gödöllő, Hungary; 2 Department of Environmental Safety and Ecotoxicology, Szent István University, Gödöllő, Hungary; 3 SeqOmics Biotechnology Ltd., Mórahalom, Hungary; 4 Institute of Plant Biology, Biological Research Centre, Hungarian Academy of Sciences, Szeged, Hungary; 5 Institute of Groundwater Ecology, Helmholtz Zentrum München, Munich, Germany; 6 Chair of Ecological Microbiology Bayreuth, Center of Ecology and Environmental Research (BayCEER), University of Bayreuth, Bayreuth, Germany

**Keywords:** *Zoogloea*, Toluene degradation, Metatranscriptomics, Biodegradation

## Abstract

*Zoogloea oleivorans*, capable of using toluene as a sole source of carbon and energy, was earlier found to be an active degrader under microaerobic conditions in aquifer samples. To uncover the genetic background of the ability of microaerobic toluene degradation in *Z. oleivorans*, the whole-genome sequence of the type strain Buc^T^ was revealed. Metatranscriptomic sequence reads, originated from a previous SIP study on microaerobic toluene degradation, were mapped on the genome. The genome (5.68 Mb) had a mean G + C content of 62.5%, 5005 protein coding gene sequences and 80 RNA genes. Annotation predicted that 66 genes were involved in the metabolism of aromatic compounds. Genome analysis revealed the presence of a cluster with genes coding for a multicomponent phenol-hydroxylase system and a complete catechol *meta*-cleavage pathway. Another cluster flanked by mobile-element protein coding genes coded a partial catechol *meta*-cleavage pathway including a subfamily I.2.C-type extradiol dioxygenase. Analysis of metatranscriptomic data of a microaerobic toluene-degrading enrichment, containing *Z .  oleivorans* as an active-toluene degrader revealed that a toluene dioxygenase-like enzyme was responsible for the ring-hydroxylation, while enzymes of the partial catechol *meta*-cleavage pathway coding cluster were responsible for further degradation of the aromatic ring under microaerobic conditions. This further advances our understanding of aromatic hydrocarbon degradation between fully oxic and strictly anoxic conditions.

## Introduction

At present, the genus *Zoogloea* (family *Zoogloeaceae*) contains five validly described species, which can be characterized as floc-forming, nitrogen-fixing bacteria. Members of the genus have been isolated from various habitats including activated sludge, soil or hydrocarbon contaminated groundwater (Xie and Yokota 2006; Shao et al. 2009; Farkas et al. 2015). Despite the fact that *Zoogloea* spp. play a crucial role in wastewater treatment by causing the flocculation of the activated sludge, limited genome sequence information is available regarding these bacteria. The first publicly available genome sequence was reported by Muller et al. (2017). Recent studies characterizing benzene- and toluene-degrading microbial communities have shown that *Zoogloea* genus-related bacteria could have an important role in the degradation of these contaminants in subsurface environments. Protein- and RNA-stable isotope probing (SIP) based analysis of an aerobic benzene-degrading microbial community revealed *Zoogloea*-related bacteria as predominant benzene-degraders (Jechalke et al. 2013). Our previous DNA- and transcriptome-SIP studies have shown that *Zoogloea oleivorans* is a highly efficient toluene degrader under microaerobic conditions (Bradford et al. 2018; Táncsics et al. 2018). We hypothesized that *Z. oleivorans* was capable of degrading toluene under microaerobic conditions due to the fact that it harbours a catechol 2,3-dioxygenase (*C23O*) gene which encodes a subfamily I.2.C-type extradiol dioxygenase enzyme (Farkas et al. 2015). Kukor and Olsen (1996) suggested that this group of extradiol dioxygenases was adapted to environments with low-oxygen concentrations, hinting at their role in ring-cleavage reactions under hypoxic conditions. On the other hand, it is known that ring-cleaving dioxygenases belonging to the same subfamily may show different oxygen affinities, as was observed in the case of chlorocatechol 1,2-dioxygenases (Balcke et al. 2008) and comparative analysis of aerobic and microaerobic BTEX-degrading enrichment cultures (Benedek et al. 2018). In the present study, to uncover the genetic background of the ability of microaerobic toluene degradation in *Z. oleivorans*, the whole-genome sequence of the type strain Buc^T^ was revealed. In addition, metatranscriptomic (non-rRNA) sequence reads originated from our previous SIP study on microaerobic toluene degradation in aquifer samples with abundant *Zoogloea* spp. (Bradford et al. 2018) were mapped on the genome.

## Materials and methods

Genomic DNA from *Zoogloea oleivorans* Buc^T^ was isolated using the DNeasy UltraClean Microbial Kit (Qiagen, Germany) according to the instructions of the manufacturer. The whole-genome sequencing was performed as described previously (Borsodi et al. 2019), briefly: Nextera Mate Pair Sample Preparation Kit (Illumina, USA) was used to generate mate-paired libraries according to the manufacturer’s protocol for gel-plus version with slight modifications. 13 µl of Mate-Paired Tagment Enzyme was used to produce a robust smear within the 7-11 kbp region. The 7-11 kbp DNA fraction was excised from the gel using the Zymoclean Large Fragment DNA Recovery kit (Zymo Research, USA) and the circularized DNA was sheared using Covaris S2. All quality measurements were performed on a TapeStation 2200 instrument (Agilent, USA). Final libraries were quantified using Qubit (ThermoFisher, USA) and sequenced on an Illumina MiSeq instrument using MiSeq Reagent Kit v2 (500 cycles) sequencing chemistry. De novo assembly and scaffolding were performed with CLC Genomics Workbench Tool v11 (Qiagen, Germany). The mate-paired reads were assembled into 107 contigs. Automatic annotation of the genome was performed by the NCBI Prokaryotic Genomes Automatic Annotation Pipeline (PGAP) v4.5 (Tatusova et al. 2016). The genome sequence of strain Buc^T^ has been deposited at the GenBank database under the WGS accession number SDKK00000000 (Bioproject: PRJNA516779; Biosample: SAMN10797634). Mapping of metatranscriptomic sequence reads (NCBI Gene Expression Omnibus accession number GSM3380032; sample name: 13CHunamp) on the de novo assembled genome of strain Buc^T^ was performed by CLC Genomics Workbench Tool v11 (Qiagen, Germany) using the following parameters: length fraction = 0.8; similarity fraction = 0.8. Phylogenetic tree was reconstructed using the maximum-likelihood algorithm using MEGA version 6.0. Tree topology and distances were evaluated by bootstrap analysis based on 1000 replicates. Graphical visualization of gene clusters was performed by using SnapGene v4.3.4.

## Results and discussion

**Fig. 1:**
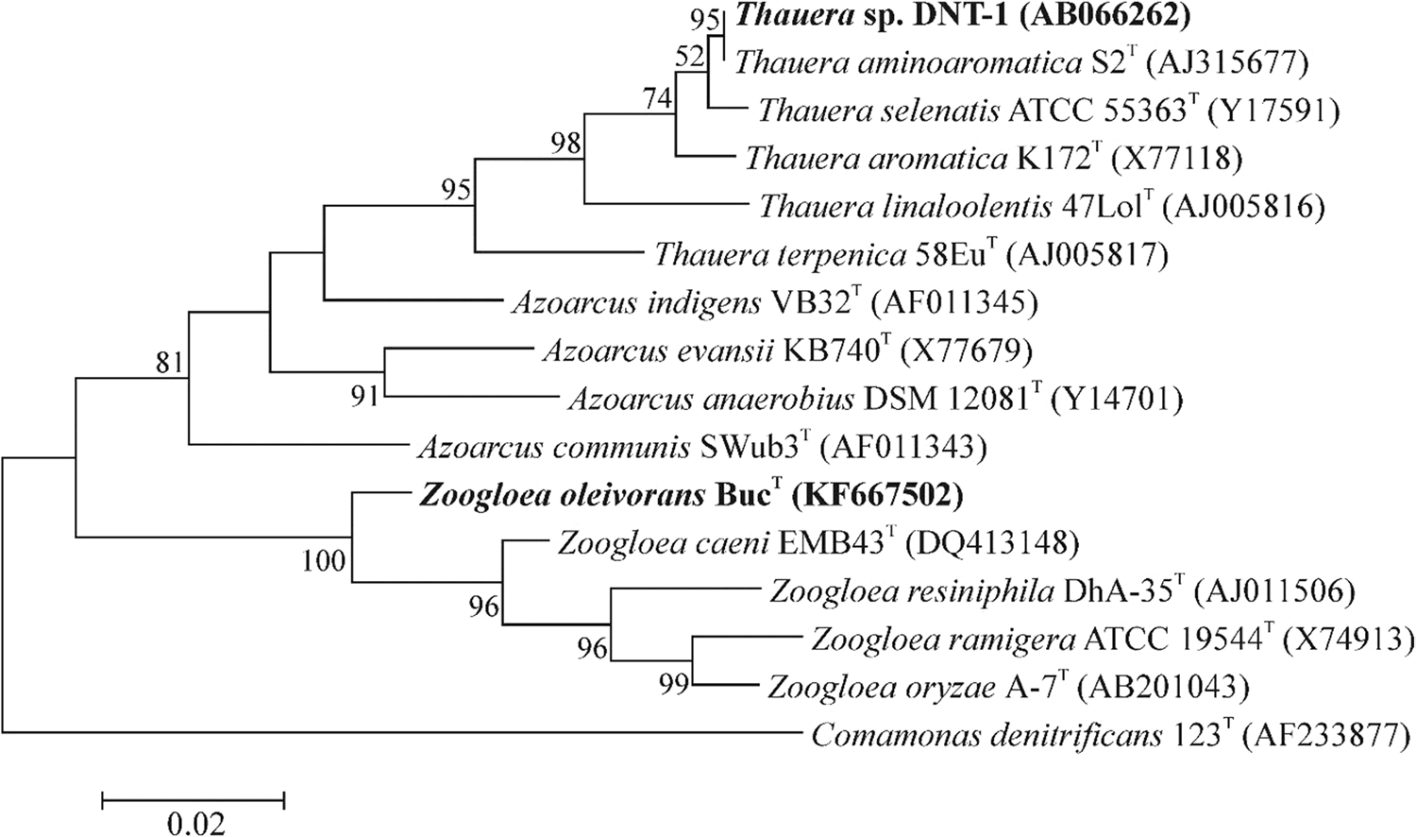
Maximum-likelihood tree based on 16S rRNA gene sequences showing the phylogenetic relationships between *Zoogloea oleivorans* Buc^T^ and related taxa including *Thaurea* sp. DNT-1 (both highlighted with boldface type). Bootstrap values are shown at nodes as percentages of 1000 replicates; only values over 50% are shown. Bar, 0.02 changes per nucleotide position

Strain Buc^T^, the type strain of *Zoogloea oleivorans,* was isolated from a petroleum-hydrocarbon contaminated environment and was previously described by us as a new member of the genus *Zoogloea* (Farkas et al. 2015). As seen on the phylogenetic tree (Fig. 1), strain Buc^T^ represents a considerably distinct lineage of the genus *Zoogloea*, and is only distantly related to typical, activated sludge inhabiting *Zoogloea* species (e.g. *Z. ramigera*).

**Fig. 2:**
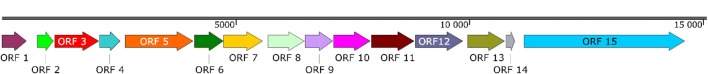
Schematic representation of the partial *meta*-cleavage pathway coding gene cluster in the genome of *Zoogloea oleivorans* Buc^T^, containing the subfamily I.2.C-type *C23O* gene. ORF 1: Tn3 family transposase; ORF 2: ferredoxin; ORF 3: I.2.C-type catechol 2,3-dioxygenase; ORF 4: heme-binding protein; ORF 5: 2-hydroxymuconic semialdehyde dehydrogenase; ORF 6: glutathione S-transferase; ORF 7: 2-hydroxymuconic semialdehyde hydrolase; ORF 8: 2-oxopent-4-enoate hydratase; ORF 9: hypothetical protein; ORF 10: SDR family oxidoreductase; ORF 11: acetaldehyde-dehydrogenase (acetylating); ORF 12: 4-hydroxy-2-oxovaleratealdolase; ORF 13: 2-oxo-3-hexendioate decarboxylase; ORF 14: 4-oxalocrotonate tautomerase family protein; ORF 15: pyruvate carboxylase

The whole-genome sequencing revealed that strain Buc^T^ has a 5,678,157 bp large genome with a G + C content of 62.5% and 5200 features (5005 protein coding genes). Annotation of the genome sequence identified that at least 66 genes affiliated with aromatic-hydrocarbon degradation. Prior to the genome sequence analysis, it was known that *Z. oleivorans* Buc^T^ harbours a catechol 2,3-dioxygenase gene, which encodes a subfamily I.2.C-type extradiol dioxygenase enzyme (Farkas et al. 2015). The genome sequence revealed that this gene is located in a gene cluster, which encodes only a partial *meta*-cleavage pathway (Fig. 2). This gene cluster is flanked by mobile-genetic elements (Tn3 family transposase upstream and an uncharacterized transposase downstream), and starts with a ferredoxin-coding gene, followed by the subfamily I.2.C-type *C23O* gene. The cluster contains 15 genes in a unique arrangement, which has not been observed before in the case of any cultured bacterium. However, this cluster was also found in the metagenome-assembled genome of an uncultivated *Rhodoferax* sp., capable of degrading sulfolane (Kasanke et al. 2019). The role of transposase mediated, partial *meta*-pathway coding gene clusters in chlorobenzene degradation was first observed and deeply studied in *Pseudomonas putida* GJ31 (Kunze et al. 2009). Since the partial *meta*-cleavage pathway coding gene cluster of strain Buc^T^ did not contain any gene encoding aromatic ring-hydroxylating dioxygenase (ARHD) enzyme, the involvement of another gene cluster in toluene degradation was assumable.

**Fig. 3:**
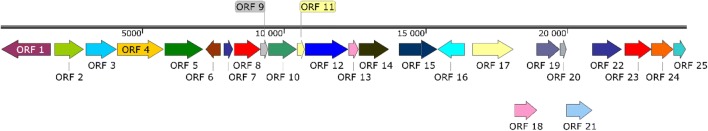
Schematic representation of the multicomponent phenol-hydroxylase coding gene cluster in the genome of *Zoogloea oleivorans* Buc^T^. ORF 1: sigma-54-dependent Fis family transcriptional regulator; ORF 2: oxidoreductase; ORF 3: aromatic ring-hydroxylating dioxygenase subunit alpha; ORF 4: DUF1302 domain-containing protein; ORF 5: DUF1329 domain-containing protein; ORF 6: YnfA family protein; ORF 7: ferredoxin; ORF 8: catechol 2,3-dioxygenase; ORF 9: phenol-hydroxylase component (DmpK); ORF 10: phenol-hydroxylase component (P1 oxygenase component, DmpL); ORF 11: phenol-hydroxylase component (P2 regulatory component, DmpM); ORF 12: phenol-hydroxylase component (P3 oxygenase component, DmpN); ORF 13: phenol-hydroxylase component (P4 oxygenase component, DmpO); ORF 14: phenol-hydroxylase component (DmpP); ORF 15: transcriptional repressor; ORF 16: XRE family transcriptional regulator; ORF 17: 2-hydroxymuconic semialdehyde dehydrogenase; ORF 18: 2-oxopent-4-enoate hydratase; ORF 19: 2-oxo-3-hexenedioate decarboxylase; ORF 20: 4-oxalocrotonate tautomerase; ORF 21: acetaldehyde-dehydrogenase (acetylating); ORF 22: 4-hydroxy-2-oxovalerate aldolase; ORF 23: catechol 2,3-dioxygenase; ORF 24: SDR family oxidoreductase; ORF 25: 4-oxalocrotonate tautomerase

**Fig. 4:**
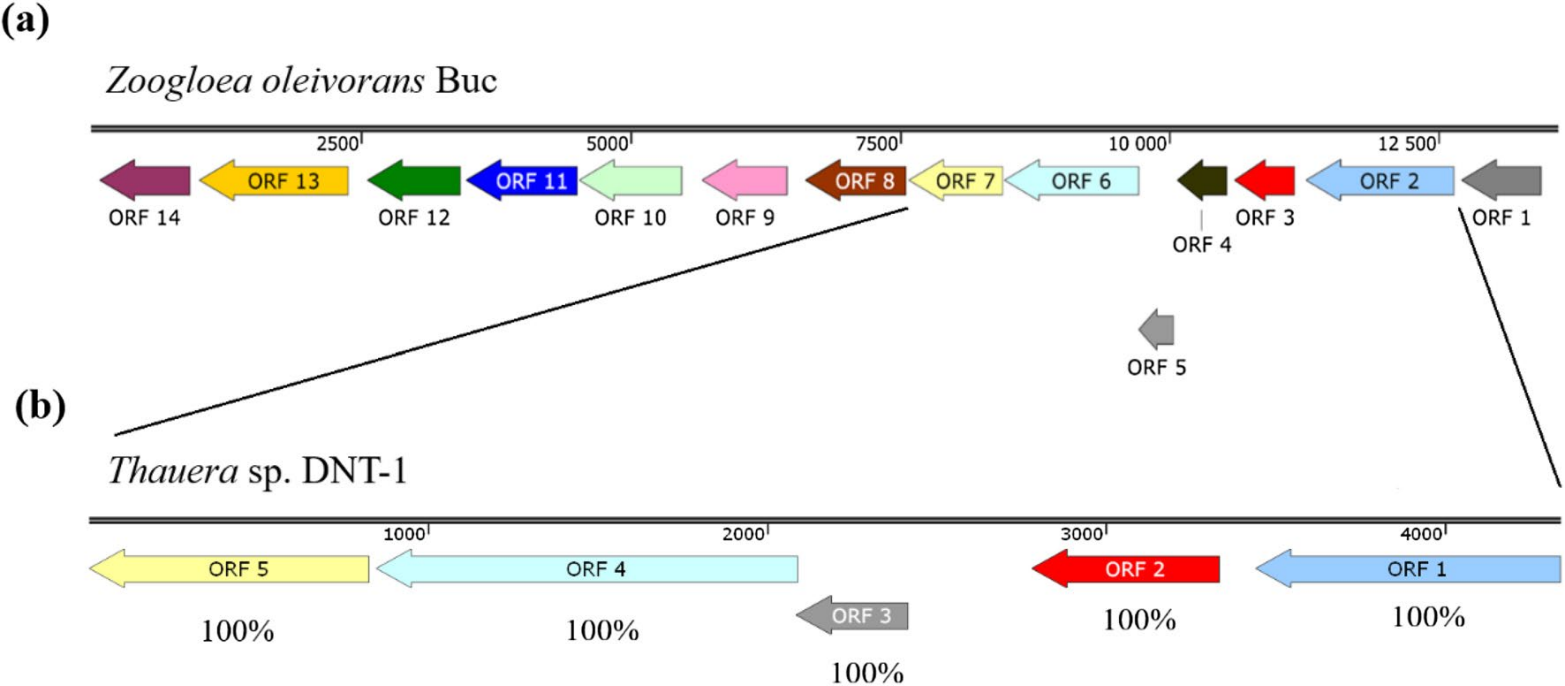
Schematic representation of the toluene-dioxygenase coding gene cluster in **a** the genome of *Zoogloea oleivorans* Buc^T^ (ORF 1: GntR family transcriptional regulator; ORF 2: aromatic ring-hydroxylating dioxygenase subunit alpha; ORF 3: 3-phenylpropionate/cinnamic acid dioxygenase subunit beta; ORF 4: hypothetical protein; ORF 5: ferredoxin; ORF 6: pyridine nucleotide-disulfide oxidoreductase; ORF 7: cis-2,3-dihydrobiphenyl-2,3-diol dehydrogenase; ORF 8: 2,3-dihydroxybiphenyl 1,2-dioxygenase; ORF 9: 2-oxopent-4-enoate hydratase; ORF 10: acetaldehyde-dehydrogenase (acetylating); ORF 11: 4-hydroxy-2-oxovalerate aldolase; ORF 12: 2-hydroxy-6-oxo-6-phenylhexa-2,4-dienoate hydrolase; ORF 13: aromatic hydrocarbon degradation protein; ORF 14: alpha/beta fold hydrolase), and **b** in the genome of *Thauera* sp. DNT-1 (GenBank accession number: AB066264) (ORF 1 and ORF 2: terminal dioxygenase iron sulphur proteins (todC1 and todC2 proteins); ORF 3 ferredoxin; ORF 4: ferredoxin reductase; ORF 5: dehyrogenase). The percentages below the ORFs indicate the similarity of nucleotide sequences to the corresponding ORF of *Zoogloea oleivorans* Buc^T^, which are depicted with the same colour

The genome sequence revealed that besides the subfamily I.2.C-type *C23O* gene, *Z. oleivorans* Buc^T^ harbours two additional *C23O* genes with different length (930 and 936 bp, respectively), which are part of a phenol-degradation gene cluster (Fig. 3). Phylogenetic analysis of these genes showed that both of them encode subfamily I.2.A.-type extradiol dioxygenases (data not shown). The shorter *C23O* gene is located upstream of genes encoding a multicomponent phenol-hydroxylase system. It shares the highest similarity (between 80-85% at nucleotide level) with *Dechloromonas*, *Zoogloea*, *Thauera* and *Azoarcus*-related *C23O* genes, which are also similarly located in phenol-degradation gene clusters. The second *C23O* gene is located close to the downstream end of the gene cluster and encodes a much more unique enzyme. Similar extradiol dioxygenase genes have been revealed so far only in the genome of *Zoogloea* sp. LCSB751, *Azoarcus communis* DSM12120 and *Methyloversatilis universalis* EHg5. Further screening of the genome for genes encoding aromatic ring-hydroxylating enzymes revealed the presence of a cluster encoding a complete *meta*-cleavage pathway. This was a biphenyl-degradation gene cluster flanked by mobile-genetic elements and contained genes of a toluene dioxygenase-like enzyme and a 2,3-dihydroxybiphenyl 1,2-dioxygenase. In this cluster the ORF 2 coded the alpha subunit (458 aa), while ORF 3 coded the beta subunit (184 aa) of the toluene dioxygenase-like enzyme (Fig. 4) which exhibited homology to todC1 and todC2 proteins of *Thauera* sp. DNT-1, respectively (100% of the amino acids are identical). It was shown that this *Thauera* strain was able to degrade toluene under both aerobic and anaerobic conditions, and in the presence of oxygen it used a toluene-dioxygenase (tod) enzyme for initial activation of the aromatic ring (Shinoda et al. 2004). The *tod* genes encoding cluster was partially recovered by Shinoda et al. (2004) and we found that all of the revealed genes in this cluster were identical with the corresponding genes in the biphenyl-degradation gene cluster of *Z. oleivorans* Buc^T^. This observation together with the fact that the biphenyl-degradation gene cluster in the genome of strain Buc^T^ was flanked by mobile-genetic elements, suggest that this gene cluster could have been spread among members of the family *Zoogloeaceae* by horizontal gene transfer (HGT).

We have previously investigated a microaerobic, ^13^C-labelled toluene-degrading enrichment culture, in which *Zoogloea oleivorans* was an abundant toluene degrader, by RNA-stable isotope probing (Bradford et al. 2018). Metatranscriptomic data (non-rRNA sequence reads of the heavy RNA fraction) derived from this enrichment study were used to reveal which of the above-mentioned gene clusters of *Z. oleivorans* were involved in degradation. The partial *meta*-cleavage pathway encoding gene cluster was found to be expressed in the enrichment, especially the subfamily I.2.C-type *C23O* and the 2-hydroxymuconic semialdehyde dehydrogenase genes (86 and 88 gene reads in the metatranscriptome, respectively, and high-RPKM values). On the other hand, genes encoding the multicomponent phenol-hydroxylase system were mostly inactive or showed low detectability (0-5 gene reads in the metatranscriptome and low RPKM values). Similary, subfamily I.2.A-type* C23O* genes, which are part of the phenol-degradation gene cluster, also showed low activity (5 and 9 gene reads, respectively, and low-RPKM values). Thus, we excluded the formation of 3-methylcatechol through 2-hydroxytoluene (*o*-cresol) and the involvement of subfamily I.2.A-type extradiol dioxygenases as possible mechanisms in the ring-cleavage reaction. However, genes of the biphenyl-degradation gene cluster, especially genes encoding the toluene-dioxygenase enzyme appeared highly expressed (115 reads altogether in the metatranscriptome). Accordingly, the formation of 3-methylcatechol through toluene-*cis*-dihydrodiol can be postulated. The phenomenon that a toluene-dioxygenase enzyme played a role in the hydroxylation of the aromatic ring under microaerobic conditions can be explained via previous observations. It has been shown for *Pseudomonas putida* F1, that the concentration of dissolved oxygen did not significantly affect the expression and longevity of toluene dioxygenase, and the strain could also grow on toluene under microaerobic conditions (Costura and Alvarez 2000). The above-mentioned *Thauera* sp. strain DNT-1 was also able to degrade toluene aerobically when only trace amount of oxygen was present in the environment (Shinoda et al. 2004). On the other hand, ring monooxygenation is usually the predominant activation mechanism of toluene degradation under microaerobic conditions, instead of dioxygenation. Thus it has been observed for toluene-degrading chemostat cultures, that *Burkholderia* (formerly *Pseudomonas*) *cepacia* strain G4, which uses a monooxygenation mechanism for toluene activation, outcompeted *Pseudomonas putida* strain F1 (using dioxygenation) under oxygen limitation (Duetz et al. 1994). A predominance of ring monooxygenation was also observed in hypoxic, toluene-degrading constructed wetlands, linked to members of the *Burkholderiaceae* and *Comamonadaceae* (Martínez-Lavanchy et al. 2015).

In summary, results of the present study provide evidence that under microaerobic conditions a toluene dioxygenase-like enzyme of *Zoogloea oleivorans* was involved in the initial activation (aromatic ring-hydroxylation) of toluene, while the subfamily I.2.C-type extradiol dioxygenase catalysed the ring-cleavage reaction. The gene clusters encoding the tod-like and the subfamily I.2.C-type extradiol dioxygenase enzymes were flanked by mobile-genetic elements, suggesting that these gene clusters were acquired by strain Buc^T^ through HGT events. Thus, the capacity of microaerobic toluene degradation seems like a mosaic encoded in the genome of *Zoogloea oleivorans* Buc^T^.
